# Commentary: Citicoline: A Food Beneficial for Patients Suffering from or Threated with Glaucoma

**DOI:** 10.3389/fnagi.2016.00194

**Published:** 2016-08-17

**Authors:** Vincenzo Parisi, Lucia Ziccardi, Gloria Roberti, Lucia Tanga, Gianluca Manni

**Affiliations:** ^1^“G.B. Bietti” Foundation for Study and Research in Ophthalmology—IRCCSRome, Italy; ^2^Department of Clinical Sciences and Translational Medicine, University of Rome Tor VergataRome, Italy

**Keywords:** citicoline, benzalkonium compounds, neuroprotection, glaucoma, eye

Dear Editor, we read with particular interest the complete review by Grieb et al. (2016).

About the paragraph “Citicoline for Glaucoma: Clinical Evidence,” we are not in agreement with the Authors' statement “The idea of topical delivery of Citicoline may not be endorsed.”

First, Grieb et al. (2016) stated that “Citicoline is water soluble and will poorly penetrate cornea.” On this topic, we recently published (Roberti et al., 2014) a study with the aim to evaluate the ability of Citicoline eye drops to reach the vitreous. The experiment was performed on five CD1 mice divided in three groups. The right eye of two mice in the Group A were treated with a solution of 1% Citicoline, 0.2% high molecular weight hyaluronic acid (HA), and 0.01% benzalkonium chloride (BAK) while the right eye of two mice in the Group B were treated with a solution of 2% Citicoline, 0.2% HA, and 0.01% BAK (OMK1®, Omikron Italia s.r.l, Italy) and one mouse in the Group C was used as control. Two drops twice-a-day for 3 days were administered. On the fourth day, after sacrificing the animal, the eyes were taken, rinsed in saline, and stored in ice. After aspirating the vitreous with a SGE Analytical Science 100 μL syringe, it was diluted with 20 μL of distilled water. Then the vitreous was centrifuged and the 50 μL of solution 50% water and 50% methanol were added to the collected supernatant and passed by vortex. For the sample analysis, liquid chromatography and mass spectrometry (LC-MS/MS) was used. The instrumentation employed consisted of triple-quadruple mass spectrometer (Applied Biosystems API 3000) equipped with a turbo ion-spray and coupled to an UPLC (Dionex Ultimate 3000 RS). Once set all instrumental and the analytical parameters to get the best conditions, the results were evaluated respect to the therapeutic range expected. This was achieved by mean of a calibration curve built by deuterated Citicoline standard solutions made at fixed concentrations. The vitreous was added with an equal volume of water/methanol solution, containing deuterated-Citicoline. Then, the sample obtained was processed though the LC-MS/MS system and between tests the machine had always done white readings to avoid contamination. Interestingly, the machine detected the molecule very well as the signal was present always at the same time and in all four transitions used. Therefore, we concluded that the molecule reached the vitreous. We also noticed that there was a difference between the concentrations 1 and 2% regarding the systemic absorption, which was zero for the lower concentration.

Second, some criticism was posed by Grieb et al. (2016) about the use of BAK, since it may “induce ocular surface changes and several other side effects.” In the cited work by Parisi et al. (2015) it is clearly reported that “Throughout the entire period of treatment with topical Citicoline, and after the 2-month period of topical Citicoline wash-out, no ocular adverse side effects were detected in any of the eyes that concluded the study.” In particular, in our study we assessed, as adverse side effects, the presence of conjunctival hyperemia, corneal micro-abrasions, conjunctival, and corneal lesions, dry eye, reduced quality, or quantity of tears and we performed the Shirmer test in all patients. None of these ocular adverse side effects have been detected in concomitance of Citicoline eye drops treatment during a period of 4 months or later during a period of 2 months of Citicoline eye drops wash-out. Furthermore, the Shirmer test was unmodified for all the tested patients at baseline and after treatment.

The lack of any observed adverse side effect, in relationship to the cohort of our enrolled patients (patients under beta-blocker monotherapy) may be ascribed to the following condition. The eye drops contained Citicoline sodium salt: 0.2 g, HA: 0.02 g, BAK 0.001 g, water for injection up to 10 ml (OMK1®, Omikron Italia, Italy). In the published review “Management and therapy of dry eye disease: report of the Management and Therapy Subcommittee of the International Dry Eye WorkShop (2007)” it is clearly reported that “The toxicity of BAK is related to its concentration, the frequency of dosing, the level or amount of tear secretion, and the severity of the ocular surface disease. In patients with mild dry eye, BAK-preserved drops are usually well tolerated when used 4–6 times a day or less.” In our study, we used BAK at the concentration of 0.01%, 3 times a day, that may be considered a very low concentration that was not responsible of any detected ocular surface changes in our previous studied patients.

In addition, in order to reach a follow-up of at least 3 years, the study with topical Citicoline was actually extended in the same patients enrolled in our previous published work (Parisi et al., 2015). The entire functional (electrophysiological and visual field) data will be available in the January 2017. At this moment, all patients completed 30 months of follow-up and any patient treated with OMK1®, showed none of the ocular adverse side effects mentioned before.

Therefore, our works (Roberti et al., 2014; Parisi et al., 2015) provide a sound evidence that (1) the Citicoline reaches the vitreous in animal model; (2) there is a proof that Citicoline may reach the vitreous and the retinal structures in patients with glaucoma: this is derived by the effects of Citicoline eye drops on the retinal increased function (neuroenhancement) with consequent improvement of the neural conduction along the visual pathways and related reduction of the mean defects values (improvement of the visual field); (3) by using BAK as vehicle for Citicoline eye drops at the concentration of 0.01% any type of ocular surface changes were not detected during our previous or actual (30 months) follow-up and thus the eye drops are well tolerated. Thus, all this lead us to believe that Citicoline eye drops can be endorsed in the management of glaucoma with the possible aim to reduce the progression of visual glaucomatous dysfunction.

## Author contributions

VP, LZ, GR, LT, GM writing and editing the manuscript.

### Conflict of interest statement

The authors declare that the research was conducted in the absence of any commercial or financial relationships that could be construed as a potential conflict of interest.
